# F252. SERVICE PROVISION FOR ULTRA-HIGH RISK FOR PSYCHOSIS: IMPLEMENTATION OF CLINICAL GUIDELINES IN ENGLAND

**DOI:** 10.1093/schbul/sby017.783

**Published:** 2018-04-01

**Authors:** Helen Stain, Lauren Mawn, Stephanie Common, Marie Pilton, Andrew Thompson

**Affiliations:** 1 Leeds Trinity University; 2 School of Psychology, Newcastle University; 3 Tees Esk Wear Valleys NHS Foundation Trust; 4 Newcastle Upon Tyne Hospitals NHS Foundation Trust; 5 Warwick Medical School, University of Warwick

## Abstract

**Background:**

Evidence from meta-analyses of randomised clinical trials show interventions for young people at ultra high risk (UHR) of developing psychosis are effective both clinically and economically. While research evidence has begun to be integrated into clinical guidelines, there is a lack of research on the implementation of these guidelines. This paper examines service provision for UHR individuals in accordance with current clinical guidelines within the National Health Service (NHS) in England.

**Methods:**

A self-report online survey was completed by clinical leaders of Early Intervention in Psychosis (EIP) teams (N=50) within the NHS across the UK.

**Results:**

Of the 50 EIP teams responding (from 30 NHS Trusts), 53% reported inclusion of the UHR group in their service mandate, with age range predominantly 14–35 years (81%) and service provided for at least 12 months (53%). Provision of services according to NICE clinical guidelines showed 50% of services offered cognitive behavioural therapy (CBT) for psychosis, and 42% offered family intervention. Contrary to guidelines, 50% of services offered antipsychotic medication. Around half of services provided training in assessment by CAARMS, psycho-education, CBT for psychosis, family work and treatment for anxiety and depression.

**Discussion:**

Despite clear evidence for the benefit of early intervention in this population, current provision for UHR within EIP services in England does not match clinical guidelines. While some argue this is due to a lack of allocated funding, it is important to note the similar variable adherence to clinical guidelines in the treatment of people with established schizophrenia.

